# Supplementary material for: Application of Sensitivity Analysis to Discover Potential Molecular Drug Targets
Source: Int J Mol Sci. 2022 Jun 13;23(12):6604. doi: 10.3390/ijms23126604 (PMC9223434; doi:10.3390/ijms23126604)
Supplement: Supplementary file 1 [file ijms-23-06604-s001.zip › ijms-1740330-supplementary.pdf]

Application of sensitivity analysis to discover potential molecular drug targets

Malgorzata Kardynska, Jaroslaw Smieja, Pawel Paszek, Krzysztof Puszynski

Supplement

Table of contents:

1. General remarks 1
2. p53 regulatory module 1
3. IFN–β signaling pathway 4
References 9

1. General remarks

The Supplement contains mathematical models that have been used as examples illustrating the method presented in the man paper. We have not introduced any changes to the notation used in the original models. Nominal values of parameters have also been assumed as in the original models.
In the following sections, models equations are presented. The variables names correspond to the molecule names and they denote concentration of those molecules.

2. p53 regulatory module

The p53/Mdm2 regulatory module, illustrated by the Fig. 2 in the main text, is described by the following equations (Puszynski et al., 2008):

(Cytoplasmic) PTEN, PTEN(t)

$$\frac{dPTEN(t)}{dt} = t_1 PTEN_t(t) - d_2 PTEN(t) \tag{S2.1}$$

Active form of PIP, $PIP_p(t)$

$$\frac{dPIP_p(t)}{dt} = a_2\left(PIP_{tot} - PIP_p(t)\right) - c_0 PTEN(t) PIP_p(y) \tag{S2.2}$$

Active Akt, $AKT_p(t)$

$$\frac{dAKT_p(t)}{dt} = a_3\left(AKT_{tot} - AKT_p(t)\right)PIP_p(t) - c_1AKT_p(t) \qquad \text{(S2.3)}$$

Cytoplasmic Mdm2, MDM(t)

$$\frac{dMDM(t)}{dt} = t_0MDM_t(t) + c_2MDM_p(t) - a_4MDM(t)AKT_p(t) -$$

$$\left(d_0 + d_1\frac{N^2(t)}{h_0^2+N^2(t)}\right)MDM(t) \qquad \text{(S2.4)}$$

Cytoplasmic phosphorylated Mdm2, $MDM_p(t)$

$$\frac{dMDM_p(t)}{dt} = a_4MDM(t)AKT_p(t) - c_2MDM_p(t) - i_0MDM_p(t) + e_0MDM_{pn}(t) -$$

$$\left(d_0 + d_1\frac{N^2(t)}{h_0^2+N^2(t)}\right)MDM_p(t) \qquad \text{(S2.5)}$$

Nuclear phosphorylated Mdm2, MDMpn(t)

$$\frac{dMDM_{pn}(t)}{dt} = i_0MDM_p(t) - e_0MDM_{pn}(t) - \left(d_0 + d_1\frac{N^2(t)}{h_0^2+N^2(t)}\right)MDM_{pn}(t) \qquad \text{(S2.6)}$$

Inactive (nuclear) p53, $P53_n(t)$

$$\frac{dP53_n(t)}{dt} = p_0 - \left(a_0 + a_1\frac{N^2(t)}{h_0^2+N^2(t)}\right)P53_n(t) + c_3P53_{pn}(t) -$$

$$\left(d_3 + d_4MDM_{pn}^2(t)\right)P53_n(t) \qquad \text{(S2.7)}$$

Active (nuclear) p53, $P53_{pn}(t)$

$$\frac{dP53_{pn}(t)}{dt} = \left(a_0 + a_1\frac{N^2(t)}{h_0^2+N^2(t)}\right)P53_n(t) - c_3P53_{pn}(t) -$$

$$\left(d_5 + d_6MDM_{pn}^2(t)\right)P53_{pn}(t) \qquad \text{(S2.8)}$$

Mdm2 transcript, $MDM_t(t)$

$$\frac{dMdm2_t(t)}{dt} = 2s_0 P_A(t) - d_7 MDM_t(t) \qquad \text{(S2.9)}$$

PTEN transcript, $PTEN_t(t)$

$$\frac{dPTEN_t(t)}{dt} = 2s_1 P_A(t) - d_8 PTEN_t(t) \qquad \text{(S2.10)}$$

Number of DSBs, N(t)

$$\frac{dN(t)}{dt} = d_{DAM} \cdot R - \frac{N(t) \cdot d_{REP} \cdot P_A(t)}{N(t) + N_{SAT} \cdot P_A(t)} + a_6 \left(\frac{A(t)}{\frac{p_1}{d_9}} \right)^4 \qquad \text{(S2.11)}$$

Apoptotic factor, A(t)

$$\frac{dA(t)}{dt} = p_1 \frac{q_3 P53_{pn}^2(t)}{q_4 + q_3 P53_{pn}^2(t)} - d_9 A(t) \qquad \text{(S2.12)}$$

where $P_A(t)$ is the probability that the gene copy is active:

$$P_A(t) = \frac{q_0 + q_1 P53_{pn}^2(t)}{q_2 + q_0 + q_1 P53_{pn}^2(t)} \qquad \text{(S2.13)}$$

To make the model easier to understand, each of the equations is preceded by a caption, related to the molecule whose concentration kinetics it describes. Used notation:

- Variables without subscripts refer to cytoplasmic content, while nuclear content is represented by subscript *n*.
- mRNA transcripts are denoted by *t* subscript.
- Phosphorylated or active form of proteins is indicated by *p* subscript.

All remaining symbols are model parameters, described in (Puszynski et al., 2008).

3. IFN–β signaling pathway

The IFN–β signaling pathway (Fig. 7 in the main text) has been introduced in (Smieja et al., 2008). The following notation is used in the model description:

- Variables refer to cytoplasmic content if no subscripts are present, while nuclear content is represented by subscript *n*.
- mRNA transcripts are denoted by *t* subscript and always refer to the cytoplasmic content; the transport of mRNA to the cytoplasm is assumed to be very fast in relation to other processes and therefore neglected in the model.
- Phosphorylated form of proteins is indicated by *p* subscript.
- Subscripts *active* and *inactive* are used to distinguish respective states of the molecules.
- Four auxiliary variables x_{auxi} have been introduced in the source paper to fit the delay between transcription factor and corresponding transcript peaks.
- $u(t)$ is a logical variable and represents the model input; $u(t) = 1$ if signal (IFN–β) is present, $u(t) = 0$ if no signal is present.

To make the model easier to understand, each of the equations that follow is preceded by a caption, related to the molecule whose concentration kinetics it describes.

Unphosphorylated cytoplasmic STAT1:

$$\frac{\mathrm{dSTAT1}}{\mathrm{dt}} = \mathrm{k_{transl}} \cdot \mathrm{STAT1_t} - \mathrm{k_{s1deg}} \cdot \mathrm{STAT1} - u(t) \cdot \frac{\mathrm{k_{s1phos}} \cdot \mathrm{STAT1}}{1 + \mathrm{k_{s1phos_{sat}}} \cdot \mathrm{STAT1}} +$$

$$+\mathrm{k_{s1dephc}} \cdot \mathrm{STAT1_p} + 2\mathrm{k_{invs1s1}} \cdot \left(\mathrm{STAT1_p}|\mathrm{STAT1_p}\right) + \mathrm{k_{invs1s2}} \cdot (\mathrm{STAT1_p}|\mathrm{STAT2_p}) +$$

$$+e_{s1} \cdot STAT1_n - i_{s1} \cdot STAT1 \qquad \text{(S3.1)}$$

Unphosphorylated cytoplasmic STAT2

$$\frac{dSTAT2}{dt} = k_{transl} \cdot STAT2_t - k_{s2deg} \cdot STAT2 - u(t) \cdot \frac{k_{s2phos} \cdot STAT2}{1 + k_{s2phos_{sat}} \cdot STAT2} +$$

$$+ k_{s2dephc} \cdot STAT2_p + k_{invs1s2} \cdot (STAT1_p|STAT2_p) +$$

$$+ e_{s2} \cdot STAT2_n - i_{s2} \cdot STAT2 \qquad \text{(S3.2)}$$

Phosphorylated cytoplasmic STAT1:

$$\frac{\mathrm{dSTAT1_p}}{\mathrm{dt}} = -\mathrm{k_{s1pdeg}} \cdot \mathrm{STAT1_p} + u(t) \cdot \frac{\mathrm{k_{s1phos}} \cdot \mathrm{STAT1}}{1 + \mathrm{k_{s1phos_{sat}}} \cdot \mathrm{STAT1}} - \mathrm{k_{s1dephc}} \cdot \mathrm{STAT1_p}$$

$$-2\mathrm{k_{s1s1}} \cdot \mathrm{STAT1_p} \cdot \mathrm{STAT1_p} - \mathrm{k_{s1s2}} \cdot \mathrm{STAT1_p} \cdot \mathrm{STAT2_p} \qquad \text{(S3.3)}$$

Phosphorylated cytoplasmic STAT2:

$$\frac{dSTAT2_p}{dt} = -k_{s2\mathrm{p}deg} \cdot STAT2_p + u(t) \cdot \frac{k_{s2phos} \cdot STAT2}{1 + k_{s2phos_{sat}} \cdot STAT2} - k_{s2dephc} \cdot STAT2_p$$

$$-k_{s1s2} \cdot STAT1_p \cdot STAT2_p \qquad \text{(S3.4)}$$

Cytoplasmic STAT1p|STAT1p complex

$$\frac{d(STAT1_p|STAT1_p)}{dt} = k_{s1s1} \cdot STAT1_p \cdot STAT1_p - k_{s1s1pdeg} \cdot (STAT1_p|STAT1_p) -$$

$$-\mathrm{i_{s1s1}} \cdot (\mathrm{STAT1_p}|\mathrm{STAT1_p}) - \mathrm{k_{invs1s1}} \cdot (\mathrm{STAT1_p}|\mathrm{STAT1_p}) \qquad \text{(S3.5)}$$

Cytoplasmic STAT1p|STAT2p complex

$$\frac{d(STAT1_p|STAT2_p)}{dt} = k_{s1s2} \cdot STAT1_p \cdot STAT2_p - k_{s1s2pdeg} \cdot (STAT1_p|STAT2_p) -$$

$$-i_{s1s2} \cdot (STAT1_p|STAT2_p) - k_{invs1s2} \cdot (STAT1_p|STAT2_p) \qquad \text{(S3.6)}$$

Cytoplasmic active IRF1 protein

$$\frac{\mathrm{dIRF1}}{\mathrm{dt}} = \mathrm{k_{transl}} \cdot \mathrm{IRF1_t} - \mathrm{k_{i1deg}} \cdot \mathrm{IRF1} - \mathrm{i_{i1}} \cdot \mathrm{IRF1} + \mathrm{e_{i1}} \cdot \mathrm{IRF1_n} \tag{S3.7}$$

Cytoplasmic inactive IRF1 protein

$$\frac{\mathrm{dIRF1}_{inactive}}{\mathrm{dt}} = -\mathrm{k_{i1deg}} \cdot \mathrm{IRF1}_{inactive} + \mathrm{e_{i1}} \cdot \mathrm{IRF1}_{inactive\_n} \tag{S3.8}$$

Unphosphorylated nuclear STAT1

$$\frac{dSTAT1_n}{dt} = i_{s1} \cdot k_v \cdot STAT1 - e_{s1} \cdot k_v \cdot STAT1_n - k_{s1deg} \cdot STAT1_n +$$

$$+2k_{invs1s1\mathrm{n}} \cdot \left(STAT1_p \middle| STAT1_p\right)_n + k_{invs1s2\mathrm{n}} \cdot \left(STAT1_p \middle| STAT2_p\right)_n +$$

$$+2k_{invys1s1} \cdot \left(Y \middle| STAT1_p \middle| STAT1_p\right)_n + k_{invs1i1} \cdot (STAT1|IRF1\ )_n -$$

$$-k_{s1i1} \cdot IRF1_{active\_n} \cdot STAT1_n \tag{S3.9}$$

Unphosphorylated nuclear STAT2

$$\frac{dSTAT2_n}{dt} = i_{s2} \cdot k_v \cdot STAT2 - e_{s2} \cdot k_v \cdot STAT2_n - k_{s2deg} \cdot STAT2_n +$$

$$+k_{invs1s2\mathrm{n}} \cdot \left(STAT1_p \middle| STAT2_p\right)_n \tag{S3.10}$$

Nuclear STAT1p|STAT1p complex

$$\frac{d\left(STAT1_p \middle| STAT1_p\right)_n}{dt} = -\left(k_{invs1s1} + k_{s1s1pdeg}\right) \cdot \left(STAT1_p \middle| STAT1_p\right)_n +$$

$$+i_{s1s1} \cdot k_v \cdot \left(STAT1_p \middle| STAT1_p\right) - k_{Ys1s1} \cdot \left(STAT1_p \middle| STAT1_p\right)_n \cdot Y_{active\_n} \tag{S3.11}$$

Nuclear STAT1p|STAT2p complex

$$\frac{d(STAT1_p|STAT2_p)_n}{dt} = -(k_{invs1s2} + k_{s1s2pdeg}) \cdot (STAT1_p|STAT2_p)_n +$$

$$+i_{s1s2} \cdot k_v \cdot (STAT1_p|STAT2_p) \quad \text{(S3.12)}$$

Active nuclear hypothetical Y phosphatase

$$\frac{\mathrm{dY_{active\_n}}}{\mathrm{dt}} = \mathrm{k_{activation}} \cdot (\mathrm{STAT1_p}|\mathrm{STAT2_p})_\mathrm{n} \cdot \mathrm{Y_{inactive_n}} -$$

$$-\mathrm{k_{Ys1s1}} \cdot (\mathrm{STAT1_p}|\mathrm{STAT1_p})_\mathrm{n} \cdot \mathrm{Y_{active_n}} + \mathrm{k_{invys1s1}} \cdot (\mathrm{Y}|\mathrm{STAT1_p}|\mathrm{STAT1_p})_\mathrm{n} \quad \text{(S3.13)}$$

Inactive nuclear hypothetical Y phosphatase

$$\frac{dY_{active\_n}}{dt} = -k_{activation} \cdot (STAT1_p|STAT2_p)_n \cdot Y_{inactive_n} \quad \text{(S3.14)}$$

Nuclear Y|STAT1p|STAT1p complex

$$\frac{\mathrm{d}(\mathrm{Y}|\mathrm{STAT1_p}|\mathrm{STAT1_p})_\mathrm{n}}{\mathrm{dt}} = \mathrm{k_{Ys1s1}} \cdot (\mathrm{STAT1_p}|\mathrm{STAT1_p})_\mathrm{n} \cdot \mathrm{Y_{active_n}} -$$

$$-\mathrm{k_{invys1s1}} \cdot (\mathrm{Y}|\mathrm{STAT1_p}|\mathrm{STAT1_p})_\mathrm{n} - \mathrm{k_{degys1s1}} \cdot (\mathrm{Y}|\mathrm{STAT1_p}|\mathrm{STAT1_p})_\mathrm{n} \quad \text{(S3.15)}$$

Nuclear active IRF1 protein

$$\frac{\mathrm{dIRF1}_n}{\mathrm{dt}} = k_v \cdot \mathrm{i_{i1}} \cdot \mathrm{IRF1} - \mathrm{k_{i1deg}} \cdot \mathrm{IRF1}_n - k_v \cdot \mathrm{e_{i1}} \cdot \mathrm{IRF1_n} -$$

$$-k_{inacti1} \cdot IRF1_n - k_{s1i1} \cdot IRF1_n \cdot STAT1_n + k_{invs1i1} \cdot (STAT1|IRF1\ )_n \quad \text{(S3.16)}$$

Nuclear inactive IRF1 protein

$$\frac{\mathrm{dIRF1}_{inactive\_n}}{\mathrm{dt}} = k_{inacti1} \cdot \mathrm{IRF1}_n - \mathrm{k_{i1indeg}} \cdot \mathrm{IRF1}_{inactive\_n} - k_v \cdot \mathrm{e_{i1\_in}} \cdot \mathrm{IRF1}_{inactive\_n}$$

(S3.17)

Nuclear STAT1|IRF1 complexes

$$\frac{d(STAT1|IRF1\,)_n}{dt} = k_{s1i1} \cdot IRF1_n \cdot STAT1_n - (k_{s1i1deg} + k_{invs1i1}) \cdot (STAT1|IRF1\,)_n \tag{S3.18}$$

IRF1 mRNA

$$\frac{\mathrm{dIRF11_t}}{\mathrm{dt}} = \mathrm{v_{i1t}} \cdot \left(\mathrm{STAT1_p}\middle|\mathrm{STAT1_p}\right)_\mathrm{n} - \mathrm{k_{degi1t}} \cdot \mathrm{IRF1_t} \tag{S3.19}$$

STAT1 mRNA

$$\frac{\mathrm{d}STAT1_t}{\mathrm{d}t} = k_{s1tprod} + v_{s1t} \cdot x_{aux4} - k_{degs1t} \cdot STAT1_t \tag{S3.20}$$

STAT2 mRNA

$$\frac{\mathrm{d}STAT2_t}{\mathrm{d}t} = k_{s2tprod} - k_{degs1t} \cdot STAT2_t \tag{S3.21}$$

TAP1 mRNA

$$\frac{\mathrm{d}TAP1_t}{\mathrm{d}t} = k_{t1tprod} + v_{t1t} \cdot (STAT1|IRF1\,)_n - k_{degt1t} \cdot TAP1_t \tag{S3.22}$$

LMP2 mRNA

$$\frac{dLMP2_t}{dt} = k_{l2tprod} + v_{l2t} \cdot (STAT1|IRF1\,)_n - k_{l2t} \cdot LMP2_t \tag{S3.23}$$

Auxiliary variables

$$\frac{\mathrm{dx_{aux1}}}{\mathrm{dt}} = \frac{1}{\mathrm{T}}\mathrm{IRF1_n} - \frac{1}{\mathrm{T}}\mathrm{x_{aux1}} \tag{S3.24}$$

$$\frac{\mathrm{dx_{aux2}}}{\mathrm{dt}} = \frac{1}{\mathrm{T}}\mathrm{x_{aux1}} - \frac{1}{\mathrm{T}}\mathrm{x_{aux2}} \tag{S3.25}$$

$$\frac{\mathrm{dx_{aux3}}}{\mathrm{dt}} = \frac{1}{\mathrm{T}}\mathrm{x_{aux2}} - \frac{1}{\mathrm{T}}\mathrm{x_{aux3}} \tag{S3.26}$$

$$\frac{dx_{aux4}}{dt} = \frac{1}{T} x_{aux3} - \frac{1}{T} x_{aux4} \quad (S3.27)$$

References

K. Puszynski, B. Hat, T. Lipniacki, Oscillations and bistability in thestochastic model of p53 regulation, J Theor Biol. 2 (254) (2008) 452–465.

J. Smieja, M. Jamaluddin, A. R. Brasier, M. Kimmel, Model-based analysisof interferon-βinduced signaling pathway, Bioinformatics 24 (20) (2008): 2363–2369.
